# Chemotherapeutic treatment of colorectal cancer in pregnancy: case report

**DOI:** 10.1186/s13256-015-0621-9

**Published:** 2015-06-13

**Authors:** Ziyad Makoshi, Claire Perrott, Khadija Al-Khatani, Fadia Al-Mohaisen

**Affiliations:** 1 Department of Surgery, College of Medicine, Prince Sattam Bin Abdulaziz University, Al-Kharj, Saudi Arabia; 2 Division of Neurosurgery, Department of Surgery, The Ottawa Hospital, Civic Campus, Ottawa, ON Canada; 3 Southampton School of Medicine, University of Southampton, Southampton, UK; 4 Department of Obstetrics and Gynecology, Women’s Specialist Hospital, King Fahad Medical City, Riyadh, Saudi Arabia; 5 Department of Pathology, King Khalid University Hospital, Riyadh, Saudi Arabia

**Keywords:** Chemotherapy, Colorectal cancer, FOLFOX, Pregnancy, Saudi Arabia

## Abstract

**Introduction:**

Colon cancer in pregnancy is uncommon. Only a small number of case reports have been published in the literature on the use of chemotherapeutic drugs during pregnancy. Reports of such cases assist clinicians in further investigating the use of chemotherapy in pregnancy.

**Case presentation:**

FOLFOX-6 was administered to a pregnant, 33-year-old Saudi woman with metastatic colon cancer from 22 to 30 weeks of gestation. Her cancer was diagnosed during her pregnancy. She tolerated the chemotherapy well and delivered a full-term baby girl with no obvious harm, and normal development was documented at her 2-year follow-up examination.

**Conclusion:**

Colon cancer during pregnancy is not easily detected and is difficult to manage. A detailed history and high clinical suspicion are needed in patients who present with symptoms and signs suggestive of malignancy. A multidisciplinary approach with patient involvement is needed to decrease morbidity and mortality caused by both treatment and the cancer in the mother and to limit side effects for the fetus. Further data and long-term follow-up are needed to better understand the potential long-term side effects of chemotherapeutic drugs on offspring.

## Introduction

Colorectal cancer (CRC) in pregnancy is uncommon but not rare [1], estimated to occur in 1 in 13,000 pregnancies [2]. The initial symptoms of CRCs overlap with expected physiological changes in pregnancy and can easily be dismissed. The management of cancer in pregnancy raises ethical and medicolegal issues, and limited literature exists to guide a management approach. Over 300 cases of colon cancer in pregnancy have been reported to date. Four individual cases of chemotherapy use during pregnancy have been reported [3–6], with five additional cases reported by the International Cancer and Pregnancy Registry (Cooper Medical School, Rowan University, Camden, NJ, USA) [7]. We report a case of a 33-year-old pregnant woman with metastatic rectal cancer treated with chemotherapy with no apparent fetal harm.

## Case presentation

A 33-year-old Saudi woman (gravida 9, para 8) presented to our emergency department at 11 weeks of gestation with abdominal pain and increased flatus. The patient had a history of bleeding per rectum for 2½ years, but she believed it was due to hemorrhoids and failed to mention it to her primary care physician. Abdominal ultrasound revealed a complex left ovarian mass (19×12cm) extending up to the epigastrium with hyperechoic solid components, and a small amount of fluid was seen in the pelvis surrounding the appendix. Her hemoglobin was 7.89g/dl, and her hematocrit was 25.2%. The result of a stool test for occult blood was positive, and biochemistry showed iron deficiency anemia. Her cancer antigen 125 (CA-125) level was elevated at 85.4kU/L. She underwent left salpingo-oophorectomy, appendectomy, and partial omentectomy by laparotomy at 13 weeks of gestation. Her pathological examination revealed mucinous adenocarcinoma of the left ovary with signet ring component and serosa positive for tumor deposits, but no lymph node invasion.

The patient was counseled regarding termination of her pregnancy for possible chemotherapy and computed tomography (CT), and she agreed to undergo these interventions. However, after a failed abortion with vaginal administration of methotrexate, she refused further intervention and decided to continue with her pregnancy. Abdominal CT showed a large rectosigmoid tumor measuring 4.2cm×4.7cm×6.3cm with mildly enlarged regional lymph nodes and four hypodense metastatic liver lesions. The patient presented 2 weeks before her next appointment with fresh bleeding per rectum. A colonoscopy revealed a large rectal mass obstructing the lumen. A pathological examination showed fragments of adenomatous mucosa with high-grade dysplasia and foci of possible invasion. Her diagnosis was established as a case of colonic *KRAS*-mutant positive adenocarcinoma with left ovarian and liver metastases. The patient’s case was discussed with maternal-fetal medicine clinicians, and a recommendation was made to start a bimonthly adjuvant FOLFOX-6 chemotherapy regimen consisting of 2-hour infusion of oxaliplatin 100mg/m^2^ and leucovorin 200mg/m^2^, 5-fluorouracil (5-FU) 400mg/m^2^ bolus, and a 5-FU 2400mg/m^2^ 46-hour infusion.

She was started on her first cycle of FOLFOX-6 chemotherapy at 22 weeks. She developed mild peripheral paresthesia in her upper and lower extremities that resolved 3 days after chemotherapy. Her CA-125 level decreased to 29.1kU/L. After five cycles of chemotherapy were completed, abdominal magnetic resonance imaging (MRI) showed an interval reduction of approximately 30% in the size of the previously seen four liver metastatic lesions. There was mild interval reduction in the size of the rectal mass, with almost stable regional lymphadenopathy. The patient completed six cycles of chemotherapy, and a decision was made to withhold further chemotherapy until after delivery. Monthly ultrasound for fetal well-being revealed normal fetal growth and development.

The patient was admitted at 38 weeks of gestation for induction of labor. She had a vaginal delivery of a healthy infant girl of birth weight 2400g with Apgar scores of 9 and 10 at 1 and 5 minutes, respectively. The patient and her infant had no immediate complications post-partum. Two weeks following delivery, the patient received her seventh cycle of FOLFOX-6 chemotherapy. The patient underwent colostomy and cholecystectomy with hepatic metastasectomy followed by six cycles of adjuvant FOLFIRI chemotherapy (folinic acid, 5-fluorouracil and irinotecan). Abdominal and chest CT with intravenous contrast performed at 15 months post-delivery revealed further hepatic lesions, pulmonary nodules and right hydronephrosis caused by a pelvic mass. A decision was made to continue with palliative chemotherapy. Follow-up at 24 months with repeat scans showed evidence of stable hepatic and pulmonary lesions with an interval increase in the size of the patient’s pelvic mass. The child was assessed in the pediatric clinic at 1 month and again at 24 months and was found to have met developmental milestones without any recognizable complications, and annual follow-ups were scheduled.

## Discussion

In a review of 205 cases of CRC in pregnancy, the mean age at diagnosis was 31 years and 85% of these cancers were below the peritoneal reflection [8], as in our patient. CRC during pregnancy is believed to affect the chances of the pregnancy’s being carried to full term with delivery of a live infant [9]. Presenting symptoms of CRC, such as abdominal pain, constipation, vomiting and anemia, may be attributed to common gastrointestinal complaints in pregnancy. Rectal bleeding may be attributed to hemorrhoids, as occurred in our patient. A diagnosis is often delayed until after the 20th week of gestation [9], and patient prognosis at presentation is usually poor [10], with most patients presenting after metastasis has occurred in Dukes stages C and D [8].

The diagnosis and staging of cancer in pregnancy can prove to be a challenge for the treating physician. Carcinoembryonic antigen levels are usually normal or mildly elevated in pregnancy [11]; therefore, they can be used in the same manner as they are in non-pregnant patients. Imaging modalities considered safe in pregnancy include diagnostic X-rays [12], ultrasounds [13], sigmoidoscopies [14] and MRI [15], whereas others pose a considerable risk to the fetus, such as CT, barium enemas and radioisotope scans, owing to the higher doses of ionizing radiation delivered to the fetus.

Radiation effects are dependent largely on both the patient size and the radiation dose; however, gestational age is not significantly linked to the fetal dose of radiation [16]. Although a single abdominal or pelvic CT scan delivers a radiation dose of approximately 24 mGy to the fetus [16], which is considered below the safety threshold [17], as a general rule, radiation imaging, as well as chemotherapeutic treatments and primary surgeries, is best delayed if possible until the second trimester, when organogenesis is complete [18, 19] and the size of the uterus allows proper surgical intervention [20].

Clinical guidelines for the management of cancer in pregnancy are available in the literature [2, 21]. However, these guidelines are based mostly on case reports of, and guidelines for, non-pregnant patients. Treatment is aimed at starting therapy for the mother as soon as possible, weighing the decision of early delivery of the fetus against completion of fetal growth and lung maturity. It is recommended that delivery occur after 32 to 35 weeks of gestation when lung maturity and fetal growth are deemed satisfactory [2]. The decision to deliver by cesarean section or vaginally depends on the presence of obstruction of the delivery canal or obstetric indications; cancer per se is not an indication to perform a cesarean section.

The recommendation for resectable CRC diagnosed before 20 weeks of gestation is surgical resection to decrease the chance of dissemination occurring later in the pregnancy [22]. Surgical resection can often be performed without the need for a hysterectomy and without disturbing the pregnancy [8]. If diagnosis occurs after 20 weeks of gestation, surgery can be postponed, allowing for fetal lung maturity to be achieved and facilitating surgical exploration [23]. If cesarean section is the decided method of delivery, colorectal tumor resection can be performed during delivery, or it can be delayed for several weeks post-partum to allow for involution of the uterus and resolution of vascular engorgement of pregnancy [23].

In our patient, a decision was made to perform a left salpingo-oophorectomy at 13 weeks of gestation because of evidence of ovarian metastases. The incidence of ovarian metastases in pregnant patients with CRC is 25% [24] and is associated with a poor prognosis [25]. Owing to the higher frequency of ovarian metastases, it is recommended that bilateral salpingo-oophorectomy be performed at the time of primary tumor resection [26, 27]; however, this approach is associated with a high incidence of spontaneous abortion, particularly in the first trimester [28], and risks as well as desire for future pregnancies should be discussed with the patient.

Although radiation therapy can be considered in the treatment of cancers located remote from the fetus, radiation doses required for treatment of cancers located in the pelvis carry a serious risk of severe or lethal effects for the fetus and are recommended to be postponed until after delivery or pregnancy termination [29].

Decisions to use chemotherapy during pregnancy are weighed against the risk of teratogenicity and the limited clinical evidence of late effects. The decision relies on the analysis of risks and benefits and clearly defined aims of using chemotherapy, which are often to prolong the gestational age of the fetus and to improve the mother’s quality of life. The evidence for selection and use of chemotherapeutic agents during pregnancy is limited, owing to the relatively low incidence of this treatment. However, FOLFOX regimes are often used in the treatment of advanced CRC, as recommended by National Comprehensive Cancer Network guidelines for patients with advanced or metastatic disease [30]. The FOLFOX regime consists of oxaliplatin, leucovorin and 5-FU. The U.S. Food and Drug Administration safety rating for the use of oxaliplatin and 5-FU during pregnancy is class D. The FOLFOX regimes are considered the gold standard for treating advanced CRC in non-pregnant patients [31].

In three case studies, authors have reported the use of FOLFOX regimes to treat CRC during pregnancy [3–5]. Gestational age at initiation of chemotherapy ranged from 13 weeks [5] to 24 weeks [4], and all reported positive fetal outcomes. The follow-up periods ranged from 11¾ months [4] to 3½ years [3], and all children were reported to be within the normal limits for height, weight and neurological development. The mothers were started on FOLFIRI regimes once delivery had occurred, and two underwent surgical resection [3, 5]. Maternal outcomes varied from being disease-free at 1 year [3], dying 1 year after diagnosis (6 months post-delivery) [4] and dying 5 months after diagnosis (16 weeks post-delivery) [5]. Only one recent case report [6] included in our present literature review described use of the FOLFIRI regime, consisting of irinotecan, leucovorin and 5-FU, during pregnancy, followed by FOLFOX post-delivery. Oxaliplatin was excluded from first-line therapy in that patient because of its neurotoxicity side effects. However, irinotecan was administered for only three cycles, which limits the results.

The low incidence of cancer during pregnancy and a patient’s decision to abort her pregnancy limit the establishment of high-level evidence for chemotherapeutic guidelines during pregnancy [32]. Congenital fetal abnormalities have been reported in a number of pregnancies in which 5-FU was administered in the first trimester because of organogenesis taking place at this point of fetal development [33–35]. This incidence can range from 14% to 19%, depending on the drug used [36]. This rate is reduced significantly to 1.3% when administration occurs in the second and third trimesters [36]. This is evident in past case reports, as well as in our patient, when chemotherapy was administered from the second trimester onward and no fetal malformations were reported [3–5]. This is concurrent with previous literature reports of healthy neonates born after being exposed to 5-FU during the second and third trimesters only [37, 38]. However, follow-up during school years in such cases would assist in evaluation of any side effects that may present later in development.

Less positive maternal outcomes were reported in two cases [4, 5]. Only one study reported the mother to be disease-free at 1 year [3]. In that patient, CRC was detected during the first trimester (12 weeks of gestation), giving some evidence of a better maternal outcome with early detection and before extensive dissemination. However, suspicion of malignancy during pregnancy remains low among physicians. A recent case report [39] involved a 37-year-old woman admitted with suspicion of a ruptured membrane who underwent an emergency cesarean section. She was discharged and returned post-operatively with persistent abdominal pain and anemia and underwent a colonoscopy that revealed a large tumor of the transverse colon.

## Conclusions

The diagnosis of CRC during pregnancy continues to be a challenge for clinicians, owing to the overlap of symptoms. A detailed history, judicious clinical suspicion and patient education cannot be overstated. The diagnostic workup can be safe for both mother and fetus, and, once a diagnosis is made, treatment should involve a multi-disciplinary team and the mother’s wishes regarding her pregnancy. Also, the benefits and risks of treatment should be discussed. Termination of pregnancy is advisable for early and aggressive treatment of CRC when possible. Available evidence supporting the use of FOLFOX chemotherapy for CRC from the second term onward, although limited, shows relatively no to minor effects on mother and fetus in the short term. However, the long-term effects of this treatment remain unknown. Follow-up of infants in later childhood and adolescence, as the central nervous system continues to develop, with additional reporting of cases is needed to establish the safety of chemotherapeutic treatment of CRC during pregnancy.

## Consent

Written informed consent was obtained from the patient for publication of this case report and accompanying images. A copy of the written consent is available for review by the Editor-in-Chief of this journal.

## Abbreviations

CA-125: cancer antigen 125
CRC: colorectal cancer
CT: computed tomography
FOLFIRI: folinic acid 5-fluorouracil and irinotecan
FOLFOX: folinic acid, 5-fluorouracil and oxaliplatin
FOLFOX-6: 2-hour infusion of oxaliplatin 100mg/m^2^ and leucovorin 200mg/m^2^, 5-fluorouracil 400mg/m^2^ bolus, and 5-fluorouracil 2400mg/m^2^ 46-hour infusion
5-FU: 5-fluorouracil
MRI: magnetic resonance imaging
